# Supplementary material for: Identification of photoperiod-induced specific miRNAs in the adrenal glands of Sunite sheep (Ovis aries)
Source: Front Vet Sci. 2022 Jul 22;9:888207. doi: 10.3389/fvets.2022.888207 (PMC9354845; doi:10.3389/fvets.2022.888207)
Supplement: Supplementary file 1 [file Data_Sheet_1.ZIP › Supplementary file/miRDeep2 file.zip!/miRDeep2 file/Novel_Structure/LP42A2/Novel_407.pdf]

Provisional ID	:	NW_014641649.1_39187
Score total	:	2.9
Score for star read(s)	:	3.9
Score for read counts	:	-4.4
Score for mfe	:	1.9
Score for randfold	:	1.6
Score for cons. seed	:	
Total read count	:	3
Mature read count	:	2
Loop read count	:	0
Star read count	:	1

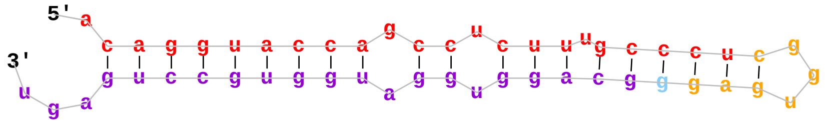

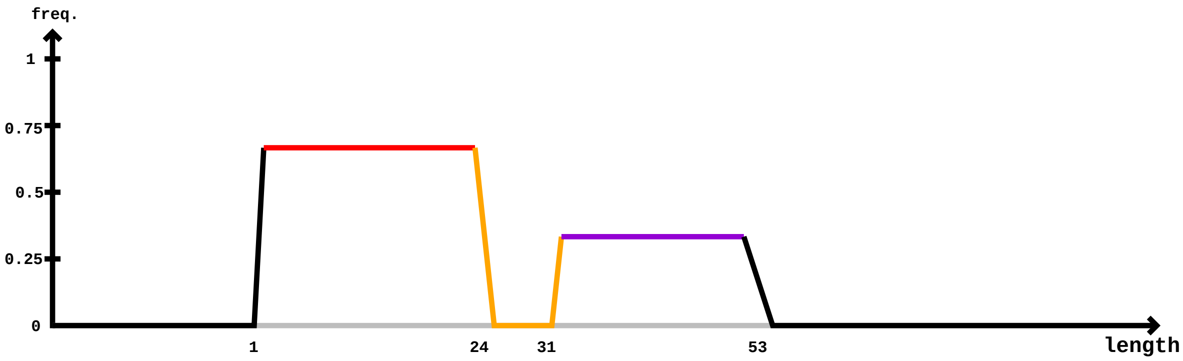


```
                    Mature                    Star
5'- gcucugcaaggaggcugaugacagguaccagccucuuugcccucggugagggcagguggauggugccugaguccuuuguccuauguccuuuccugcuuuccaaaggccacucu  -3'   obs
    gcucugcaaggaggcugaugacagguaccagccucuuugcccucggugagggcagguggauggugccugaguccuuuguccuauguccuuuccugcuuuccaaaggccacucu         exp
    .....((((((.((((.....(((((((((.((.(((.((((((...))))))))).)).)))))))))))))))))))....((.(((((..........))))).))....    reads    mm    sample
    ....................acagguaccagccucuuugcccu......................................................................    2        0     seq
    ...................................................gcagguggauggugccugagu.........................................    1        0     seq
```
